# Risk for pneumonia requiring hospitalization or emergency room visit according to delivery device for inhaled corticosteroid/long-acting beta-agonist in patients with chronic airway diseases as real-world evidence

**DOI:** 10.1038/s41598-019-48355-2

**Published:** 2019-08-19

**Authors:** Ju-Hee Park, Yunjung Kim, Seongmi Choi, Eun Jin Jang, Jimin Kim, Chang-Hoon Lee, Jae-Joon Yim, Ho-il Yoon, Deog Kyeom Kim

**Affiliations:** 1 Division of Pulmonary and Critical Care Medicine, Department of Internal Medicine, Seoul Metropolitan Government-Seoul National University Boramae Medical Center, Seoul, Republic of Korea; 2 National Evidence-based Healthcare Collaborating Agency, Seoul, Republic of Korea; 3 National Health Insurance Service, Wonju, Republic of Korea; 4 Department of Information Statistics, College of Natural Science, Andong National University, Andong, Republic of Korea; 5 Department of Health Policy and Hospital Management, Graduate School of Public Health, Korea University, Seoul, Republic of Korea; 6 Division of Pulmonary and Critical Care Medicine, Department of Internal Medicine, Seoul National University Hospital, Seoul, Republic of Korea; 7 Department of Internal Medicine, Seoul National University College of Medicine, Seoul, Republic of Korea; 8 Division of Pulmonary and Critical Care Medicine, Department of Internal Medicine, Seoul National University Bundang Hospital, Seongnam-Si, Republic of Korea

**Keywords:** Asthma, Chronic obstructive pulmonary disease

## Abstract

A fixed-dose combination of inhaled corticosteroid and long-acting beta agonist (ICS/LABA) may increase the risk of pneumonia in patients with chronic airway diseases including chronic obstructive pulmonary disease and asthma. Although lung deposition of ICS/LABA is dependent on the inhaler device and inhalation technique, there have been few studies comparing the risk for pneumonia according to the type of device used to deliver ICS/LABA in real-world practice. A retrospective cohort study was performed using the National Health Insurance Database of the Korean Health Insurance Review & Assessment Service. New users who began ICS/LABA were selected and followed-up 180 days after ICS/LABA initiation. The risk for pneumonia requiring emergency room (ER) visit or admission was compared according to inhaler device used—pressurized metered-dose inhaler (pMDI) or dry powder inhaler (DPI)—after individual exact matching (1:5). Among the eligible cohort of 245,477 new ICS/LABA users, 7,942 patients who used pMDI only were matched with 39,690 patients who used DPI only. The incidence of pneumonia was higher in the pMDI group (1.6%) than the DPI group (1.1%); the adjusted hazard ratio (HR) for pneumonia was 1.6 (95% CI 1.3–2.0; p < 0.0001). In subgroup analyses, a significantly higher risk for pneumonia was found in the pMDI group compared with the DPI group regardless of the presence of history of pneumonia (HR 1.7 [95% CI 1.2–2.3]; p = 0.002), COPD (HR 1.6 [95% CI 1.2–2.0]; p = 0.0007), or asthma (HR 1.6 [95% CI 1.2–2.2]; p = 0.0008). In analyses of real-world data, pMDI users incurred a higher risk for pneumonia requiring hospitalization or ER visit compared with DPI users.

## Introduction

A fixed-dose combination (FDC) of inhaled corticosteroid (ICS) and long-acting beta agonist (LABA) is one of the most frequently used forms of inhaled respiratory medication for chronic airway diseases, including chronic obstructive pulmonary disease (COPD) and asthma^1,2^. Although it has been established that an FDC of ICS/LABA can provide symptomatic relief and prevent acute exacerbation of COPD^3,4^ and asthma^5^, it can increase the risk for pneumonia which is one of the major causes of acute exacerbation, especially in COPD^6,7^. Recently, the ICS fluticasone propionate (FP) was reported to suppress innate and acquired anti-viral immune responses leading to increased pulmonary bacterial load and mucus production during exacerbations of COPD^8^.

In addition to treatment-independent risk factors, such as the severity of COPD and patient age^7^, intrinsic properties or cumulative dose of ICS are associated with a higher risk for pneumonia^9,10^. Most importantly, many studies have repeatedly demonstrated that cumulative dosage and duration of exposure are key factors that increase the risk for pneumonia in patients with chronic airway diseases^1,9^. In terms of cumulative dose in the lung, the proportion of lung deposition of ICS can be different according to individual characteristics of inhaler and inhalation techniques^11,12^, and may affect the risk for development of pneumonia in ICS/LABA users with chronic respiratory diseases^13^.

The two most commonly used devices used to deliver combined ICS/LABA in clinical practice to achieve effective delivery to the lungs are pressurized metered-dose inhalers (pMDIs) and dry powder inhalers (DPIs)^14^. Several previous studies have compared the effectiveness and possible complications between the two types of inhaler^15,16^; nevertheless, definitive conclusions are lacking due to insufficient data, especially in real-world practice. In a recent study comparing the effectiveness of FP/salmeterol (SAL) combination therapy via pMDI versus DPI inhaler in reducing exacerbation(s) in COPD, pneumonia risk was not different according to inhaler type^13^. However, in this historical matched cohort study, the number of pMDI users was very small and pneumonia risk was not a primary outcome measure of the analysis, and FP/SAL via pMDI is not licensed for the treatment of COPD. Therefore, the results regarding this issue were inconclusive.

Therefore, we conducted an analysis using the Korean Nation-wide Health Insurance Database of Health Insurance Review and Assessment Service (HIRA, Seoul, Republic of Korea) to compare the risk for pneumonia requiring hospitalization in patients with chronic airway diseases such as COPD, asthma, bronchiectasis, and tuberculosis-destroyed lung who used ICS/LABA FDC inhalers in real-world practice.

## Methods

### Data source

Data from the HIRA database, which includes 50.9 million South Koreans from the National Health Insurance and National Medical Aid databases, were used. The HIRA database contains information regarding demographics and all medical services rendered, along with diagnostic codes (*International Statistical Classification of Diseases and Related Health Problems*, 10^*th*^ *Revision*, [ICD-10]) and all medications prescribed. Missing or out of range values in key fields, such as drug name, quantity, date dispensed, and duration, only account for <0.5% of all records. This study was approved by the Ethics Review Committee of the National Evidence-based Healthcare Collaborating Agency, Seoul, Republic of Korea. Given the retrospective nature of the present study and the use of anonymized patient data/records, requirements for informed consent were waived.

### Study design and population

A retrospective cohort study was performed to compare the risk for pneumonia requiring ER visitation or admission according to inhaler device in the HIRA database. The source population consisted of all individuals who were dispensed inhaled respiratory drugs for ≥30 days between January 1, 2009, and December 31, 2011.

The index date was defined as the date of first use of inhaled respiratory drugs. From this cohort, the following patients were excluded: those with prescriptions for inhaled respiratory drugs for ≥30 days during the year before the index date; those who were prescribed inhaler medication other than ICS/LABA within 180 days of the index date; and those who were ≤20 or >100 years of age.

**Figure 1:**
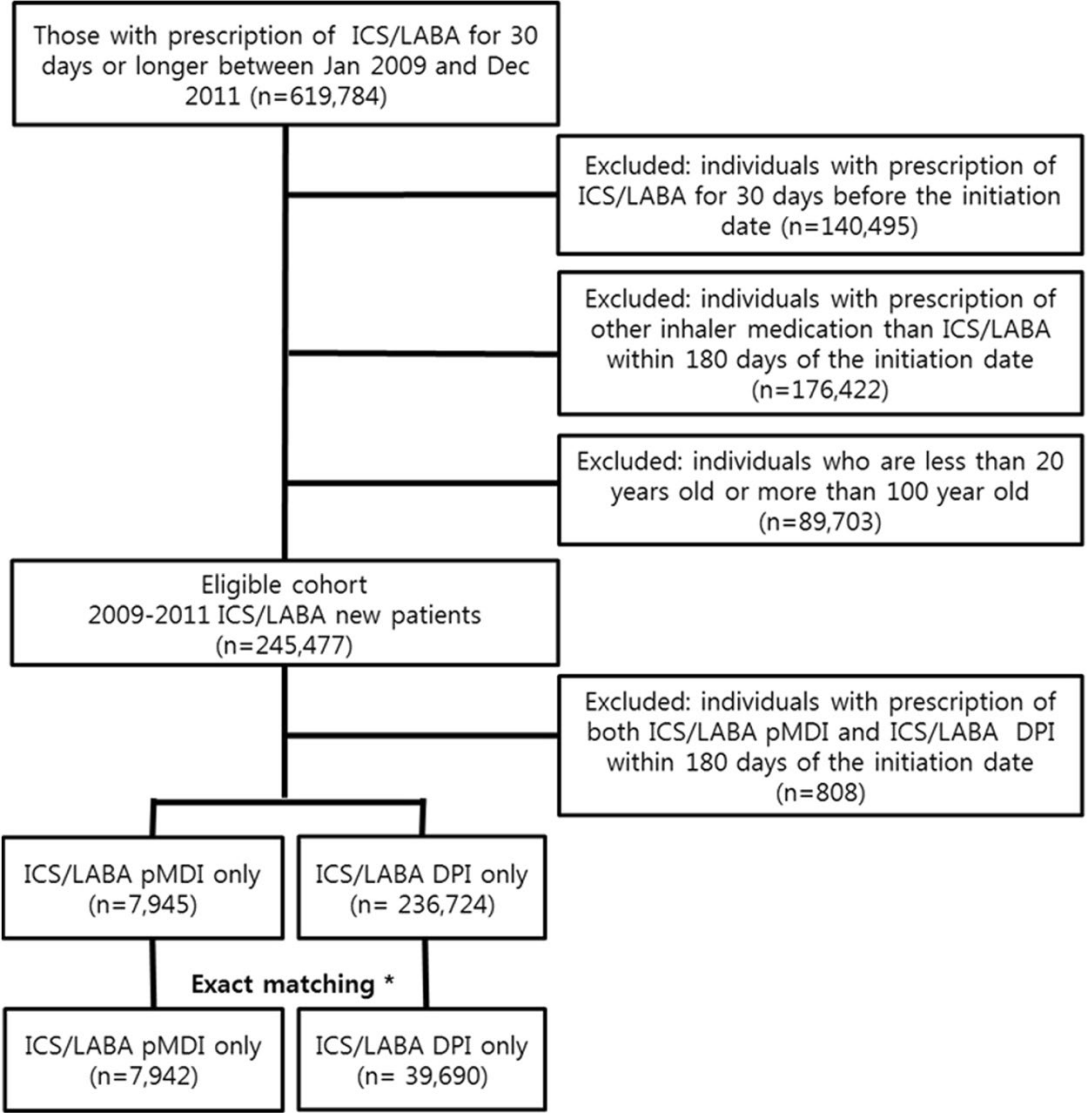
A flowchart for selecting the study population. *Exact matching was performed using the covariates such as age, sex, pneumonia in the past year, history of respiratory disease, and the Charlson comorbidity index. ICS/LABA: Inhaled Corticosteroids/Long acting beta agonist; pMDI: pressurized metered-dose inhaler; DPI: Dry power inhaler.

The inhaled drug included was a combination of an ICS/LABA (budesonide [BUD]/formoterol [FOR] or FP/SAL). ICS/LABA was classified as ICS/LABA delivered via DPI or pMDI devices. In the enrollment period, only BUD/FOR (as DPI formulation) or FP/SAL (as a pMDI or DPI formulation) FDC inhalers were available in Korea. Inhaler users were defined as individuals who used inhaled drugs for ≥30 days during one year; patients taking respiratory drugs requiring a nebulizer were excluded from this study. A flowchart detailing patient selection flow is presented in Fig. 1.

### Individual exact matching between DPI and pMDI group

Individual exact matching was performed to select a comparable DPI-using patient for each pMDI-using patient (7,945 patients). Individuals using a DPI were selected from among 236,724 patients who were prescribed DPI inhalers only. Each pMDI-using patient was matched with up to five DPI-using patients based on covariates such as age (±5 years), sex, whether they had a history of pneumonia in the past year, history of respiratory disease such as tuberculosis-lung (ICD-10 code B90), bronchiectasis (ICD-10 code J47), asthma (ICD-10 codes J45-46), and Charlson Comorbidity Index (CCI) score in the year before the index date. The standardized difference (STD) was used to assess the covariate balance before and after matching. The corresponding covariate was defined to be unbalanced when the difference in STD was ≥10%^17^.

### Definition of pneumonia

Within the eligible cohort, pneumonia was defined according to ICD-10 diagnoses (ICD-10 codes J12-J18) after the index date of initiation of inhaled respiratory drugs. The date of the first assignment of the pneumonia ICD-10 code was defined as the event date. The event was defined when the patient was diagnosed with pneumonia (ICD-10 codes J12-18) according to records of ER visitation or admission.

### Covariates of assessment for risk for pneumonia

Covariates used to assess for pneumonia risk included comorbidities, health care utilization, concomitant other medications, and ICS/LABA cumulative dose. Comorbidities included diabetes (ICD-10 code E10-E14), malignancy (ICD-10 code C00-C97), chronic kidney disease or dialysis (ICD-10 codes N17-N19), stroke (ICD-10 codes I60-I69), heart disease (ICD-10 codes I20-I25, I50), and liver disease (ICD-10 codes K70-K77). Health care utilization data, such as number of hospitalizations and outpatient ER visits, were used to adjust severity of disease. Concomitant medications included systemic corticosteroids and immunosuppressive agents including tissue necrosis factor (TNF)-alpha. Adherence to medication such as medication possession ratio was not analyzed. However, the prescription days and the cumulative doses of ICS/LABA were considered and analyzed. Also those factors were used as adjustment covariables to minimize the bias associated with the difference of exposure to medication.

### Statistical analysis

Baseline characteristics are summarized according to descriptive statistics such as proportion, mean, standard deviation (SD), median, first quartile (Q1), and third quartile (Q3). Continuous variables were also summarized into the appropriate categories based on their distributions. Statistical significance was determined using the independent t-test for continuous variables and the chi-squared-test for categorical variables.

The association between inhaler device and pneumonia risk was investigated using a Cox proportional hazards regression model. Unadjusted hazard ratio (HR) and adjusted HR (aHR) are presented with corresponding 95% confidence interval (CI). Subgroup analyses assessing the risk for pneumonia in DPI and pMDI users were performed according to history of pneumonia, COPD and asthma. All statistical analyses were performed using SAS version 9.2 (SAS Institute, Cary, NC, USA); p < 0.05 was considered to be statically significant.

## Results

### Patient selection and baseline characteristics

In total, 619,784 individuals with prescriptions for inhaled FDC ICS/LABA for ≥30 days between January 1, 2009, and December 31, 2011, were identified from the database. Of these individuals, 374,307 were excluded for the following reasons (Fig. 1): previous prescriptions for inhaled respiratory drugs for ≥30 days during the year before the current initiation of inhaled respiratory medication (n = 140,495); prescribed inhaler medication other than ICS/LABA within 180 days of the initiation date (n = 176,422); and <20 years or, >100 years of age (n = 89,703). Finally, an eligible cohort of 245,477 new users of FDC ICS/LABA was identified. However, 808 patients who were prescribed both ICS/LABA pMDI and DPI within 180 days of the initiation date were also excluded. As a result, 7,945 and 236,724 individuals were prescribed only the pMDI and DPI type ICS/LABA inhaler, respectively, during the study period. After exact matching according to age, sex, history of pneumonia, history of respiratory disease and CCI, 7,942 individuals used only the pMDI inhaler for ICS/LABA, and 39,690 matched patients who used only DPI inhaler for ICS/LABA were included in the final analysis (Fig. 1). The proportion of FP/SAL DPI was 75.3% of all ICS/LABA DPIs in unmatched population.

The baseline characteristics of the study population before and after exact matching are listed in Table 1. Before matching, DPI users were younger and had a lower prevalence of non-respiratory comorbidities, and the prevalence of asthma was higher in pMDI users. However, after proper matching, there was no difference in main baseline characteristics between the groups, except for the prevalence of chronic renal disease and liver disease (Table 1). In the matched groups, females were slightly more dominant (59.3%), and the median age of the patients was 57 and 59 years for the DPI and pMDI groups, respectively. Approximately 4.4% of patients had a history of pneumonia in the previous year. Among the chronic respiratory diseases, asthma was most common (58.5%), followed by COPD (33.1%) and bronchiectasis (3.0%). The median CCI was 2, and the most common comorbidity was liver disease(s) and diabetes mellitus.

**Table 1:** Baseline characteristics of patients using ICS/LABA.

Variable	Before matching				After matching*			
	pMDI (N = 7,945)	DPI (n = 236,724)	*P*-value	STD (%)	pMDI (N = 7,942),	DPI (n = 39,690),	*P*-value	STD (%)
Age								
Mean ± SD	57.1 ± 17.2	53.1 ± 16.2	<0.0001	23.9	57.1 ± 17.2	55.7 ± 16.4	<0.001	8.3
Median(Q1, Q3)	59 (44, 71)	54 (40, 66)			59 (44, 71)	57 (43, 69)		
Sex (male, %)	3,237 (40.7%)	91,971 (38.9%)	0.0007	3.9	3,236 (40.7%)	16,167 (40.7%)	1.0	0.0
Pneumonia in the past year	350 (4.4%)	7,080 (3.0%)	<0.0001	7.5	347 (4.4%)	1,717 (4.3%)	0.863	0.5
Respiratory disease			<0.0001				1.0	
Asthma	4,644 (58.5%)	148,772 (62.8%)		9.0	4,644 (58.5%)	23,215 (58.5%)		0.0
COPD	2,625 (33.1%)	64,583 (27.3%)		12.6	2,625 (33.1%)	13,123 (33.1%)		0.0
Bronchiectasis	241 (3.0%)	8,608 (3.6%)		3.4	241 (3.0%)	1,201 (3.0%)		0.0
Tuberculosis destroyed lung	88 (1.1%)	2,467 (1.0%)		0.6	88 (1.1%)	439 (1.1%)		0.0
Others	346 (4.3%)	12,294 (5.2%)		3.9	344 (4.3%)	1,712 (4.3%)		0.0
Charlson comorbidity index								
Mean ± SD	2.4 ± 1.8	2.14 ± 1.62	<0.0001	17.6	2.4 ± 1.8	2.38 ± 1.76	0.381	1.1
Median (Q1, Q3)	2 (1, 3)	2 (1, 3)			2 (1, 3)	2 (1, 3)		
Comorbidities								
Diabetes	1,631 (20.5%)	39,176 (16.5%)	<0.0001	10.3	1,630 (20.5%)	7,967 (20.1%)	0.3606	1.0
Liver disease	1,649 (20.8%)	47,177 (19.9%)	0.07	2.1	1,648 (20.8%)	8,921 (22.5%)	0.0007	4.1
Heart disease	1,197 (15.1%)	27,431 (11.6%)	<0.0001	10.2	1,195 (15.0%)	5,610 (14.1%)	0.034	2.6
Stroke	702 (8.8%)	15,347 (6.5%)	<0.0001	8.9	701 (8.8%)	3,311 (8.3%)	0.156	1.8
Malignancy	445 (5.6%)	9,818 (4.1%)	<0.0001	6.8	445 (5.6%)	2,139 (5.4%)	0.4425	0.9
Chronic renal disease	138 (1.7%)	2,708 (1.1%)	<0.0001	5.0	137 (1.7%)	565 (1.4%)	0.0418	2.4
**Health care utilization**								
Number of hospitalization								
Mean ± SD	0.42 ± 1.14	0.33 ± 1.01	<0.0001	8.4	0.42 ± 1.13	0.39 ± 1.13	0.1198	2.7
Median (Q1, Q3)	0(0, 0)	0(0, 0)			0(0, 0)	0(0, 0)		
Number of outpatient visit								
Mean ± SD	30.7 ± 31.5	28.6 ± 27.9	<0.0001	7.1	30.8 ± 31.5	31.1 ± 29.7	0.349	1.0
Median (Q1, Q3)	22 (12, 39)	21 (11, 36)			22 (12, 39)	23 (12, 40)		

^*^Exact matching was performed using the covariates such as age, sex, pneumonia in the past year, history of respiratory disease, and the Charlson comorbidity index. Student’s T test was conducted for continuous values and Chi-squared test was conducted for categorical values in assessing P-value.

ICS/LABA: Inhaled Corticosteroids/Long acting beta agonist; pMDI: pressurized metered-dose inhaler; DPI: Dry power inhaler; COPD: Chronic obstructive pulmonary disease, STD: Standardization difference.

Regarding exposure to ICS/LABA in each group, the mean number of prescription days was higher for DPI than pMDI users (49.8 ± 35.02 days versus[vs] 41.0 ± 26.43 days, respectively; p < 0.0001), while their median value was not different. When the number of patients who were prescribed ICS/LABA for 30, 60, or >60 days were stratified, the distribution pattern was different, and the DPI group had a larger proportion of patients whose prescription was a longer duration than pMDI group, although patients prescribed for <30 days were predominant in both groups (Table 2).

**Table 2:** Exposure to ICS/LABA and concomitant immunosuppressives in study group.

Variable	Before matching				After matching			
	pMDI (N = 7,945)	DPI (N = 236,724)	*P*-value	STD (%)	pMDI (N = 7,942)	DPI (N = 39,690)	*P*-value	STD (%)
ICS/LABA prescription (days)								
Mean ± SD	41.0 ± 26.45	48.8 ± 34.3	<0.0001	25.2	41.0 ± 26.4	49.8 ± 35.0	<0.0001	28.4
Median (Q1, Q3)	30 (30, 30)	30 (30, 60)			30 (30, 30)	30 (30, 60)		
Patients prescribed with ICS/LABA for following days (n, %)	<0.0001				<0.0001			
0< ≤ 30 days	6,253 (78.7%)	160,724 (67.9%)		24.6	6,252 (78.7%)	26,337 (66.4%)		27.8
30< ≤ 60 days	945 (11.9%)	35,833 (15.1%)		9.4	944 (11.9%)	6,267 (15.8%)		11.3
60 days<	747 (9.4%)	40,167 (17.0%)		22.6	746 (9.4%)	7,086 (17.9%)		25.0
ICS/LABA cumulative dose								
Mean ± SD	31,461.3 ± 24155.91	21,956.6 ± 19547.8	<0.0001	43.3	31,456.08 ± 24,152.79	22,568.93 ± 20,284.58	<0.0001	39.8
Median (Q1, Q3)	30,000 (30,000, 30,000)	15,000 (15,000, 30,000)			30,000 (15,000, 30,000)	15,000 (15,000, 30,000)		
Patients exposed to following cumulative dose of ICS/LABA (n, %)	<0.0001				<0.0001			
0< ≤ 1,5000 dose	329 (4.1%)	48,895 (20.7%)		52.0	328 (4.1%)	8,000 (20.2%)		50.8
1,5000< ≤ 3,0000 dose	2,535 (31.9%)	126,424 (53.4%)		44.5	2,535 (31.9%)	20,789 (52.4%)		42.4
3,0000 dose <	5,081 (64.0%)	61,405 (25.9%)		25.9	5,079 (64.0%)	10,901 (27.5%)		78.7
Concomitant other medication								
Systemic-corticosteroids prescription days								
Mean ± SD	40.5 ± 116.3	36.3 ± 103.0	0.0015	3.8	40.5 ± 116.3	38.8 ± 109.2	0.25	1.5
Median (Q1, Q3)	10 (10, 31)	10 (10, 30)			10 (1, 31)	11 (2, 31)		
Other immunosuppressives including TNF-alpha (n, %)	65 (0.8%)	1,592 (0.7%)	0.12	1.2	64 (0.8%)	299 (0.8%)	0.62	0.0

Student’s T test was conducted for continuous values and Chi-square test was conducted for categorical values in assessing P-value. ICS/LABA: Inhaled Corticosteroids/Long acting beta agonist; pMDI: pressurized metered-dose inhaler; DPI: Dry power inhaler, STD: Standardization difference.

The cumulative dose of ICS/LABA was calculated because it was higher in pMDI users than in DPI users (31,456 ± 24,152 vs 22,568 ± 20,284, respectively; p < 0.0001). Additionally, the number of patients who were exposed to a higher cumulative dose of ICS/LABA was greater in the pMDI group than in the DPI group (Table 2). Therefore, the cumulative dose of ICS/LABA was included in the adjustment of covariates for assessment of pneumonia risk in the following steps.

The duration of exposure to systemic steroids and other immunosupressives, including TNF-alpha, was not different between the groups.

### Impact of ICS/LABA delivery device on the risk for pneumonia

Pneumonia events occurred in 131 (1.6%) patients among new pMDI users for ICS/LABA, while 451 (1.1%) patients experienced pneumonia in new user for DPI. The HR for pneumonia in the pMDI group compared with DPI group was 1.46 (95% CI 1.2–1.8; p < 0.001). After adjusting for covariates including comorbidities, healthcare utilization, concomitant other medications, and cumulative dose of ICS/LABA, the result remained consistent (Table 3). The risk for pneumonia according to devices was not changed even after adjusting with prescription days as a categorical (HR 1.3 (95% CI 1.1–1.6), p = 0.013) or continuous variable (HR 1.3 (95% CI 1.1–1.6), p = 0.006).

**Table 3:** Risk of pneumonia according to ICS/LABA device type.

Cases (n, %)	ICS/LABA device type		Unadjusted		Adjusted*	
	pMDI	DPI	Hazard ratio (HR)**	P value	HR** (95% CI)	p-value
Total	7,942	39,690	1.5 (1.2,1.8)	0.0001		
Pneumonia (+)	131 (1.6%)	451 (1.1%)			1.6 (1.3, 2)	<0.0001
Pneumonia (−)	7811 (98.4%)	39,239 (98.9%)				

ICS/LABA: Inhaled Corticosteroids/Long acting beta agonist; pMDI: pressurized metered-dose inhaler; DPI: Dry power inhaler.

*Adjustment covariates for risk assessment of pneumonia were comorbidities, healthcare utilization, concomitant other medications, and cumulative dose of ICS/LABA usage.

**Hazard ratio was calculated as the ratio of risk of pneumonia in pMDI group to DPI group.

**Figure 2:**
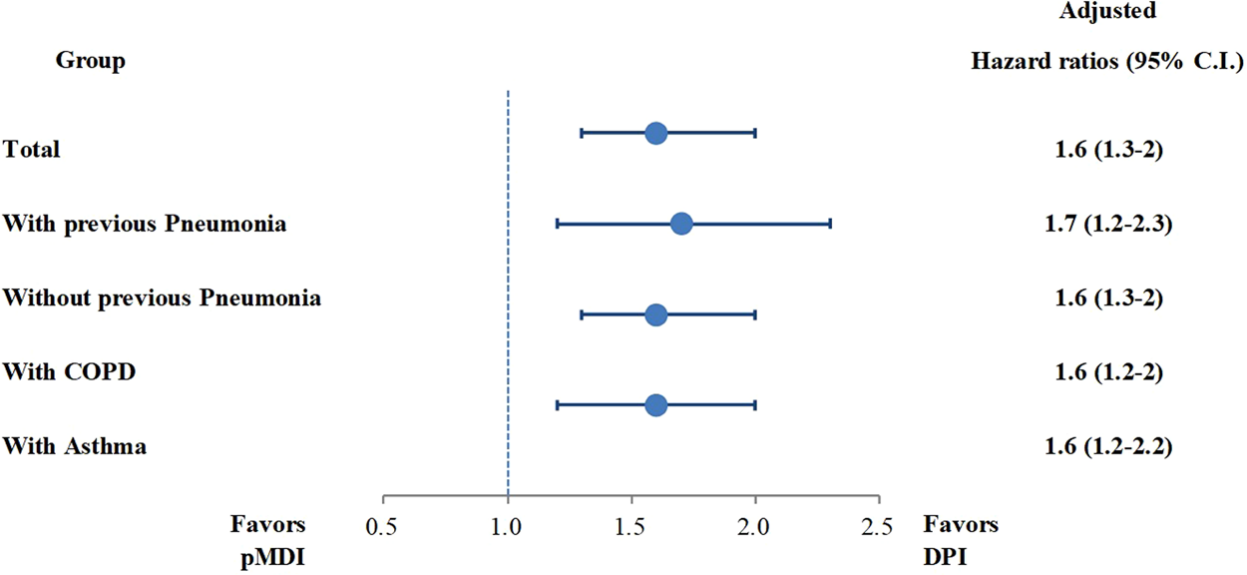
Hazards ratio for pneumonia among new pMDI user for ICS/LABA compared with the DPI group. Schematic presentation of hazards ratio of pneumonia in subjects using pMDI when compared with those using DPI. Bar presents confidence of interval with 95% significance. ICS/LABA: Inhaled Corticosteroids/Long acting beta agonist; pMDI: pressurized metered-dose inhaler; DPI: Dry power inhaler; COPD: Chronic obstructive pulmonary disease; C.I.: Confidence of interval.

When the groups were stratified according to history of pneumonia, the risk for pneumonia was consistently increased in the pMDI group compared with DPI group, irrespective of history of pneumonia (Table 4 and Fig. 2).

**Table 4:** Subgroup analysis for risk assessment of pneumonia according to ICS/LABA device use.

Cases (n, %)	ICS/LABA device type		Unadjusted		Adjusted*	
	pMDI	DPI	HR** (95% CI)	p-value	HR** (95% CI)	p-value
With past history of pneumonia	(N = 347)	(N = 1,717)				
Pneumonia (+)	40 (11.4%)	122 (7.1%)	1.65 (1.2, 2.3)	0.0023	1.7 (1.2, 2.3)	0.0022
Pneumonia (−)	307 (88.6%)	1,595 (92.9%)				
Without past history of pneumonia	(N = 7,595)	(N = 37,973)				
Pneumonia (+)	91 (1.2%)	266 (0.7%)	1.67 (1.35, 2.06)	<0.0001	1.6 (1.3, 2)	<0.0001
Pneumonia (−)	7504 (98.8%)	37,707 (99.3%)				
COPD	(N = 2,625)	(N = 13,123)				
Pneumonia (+)	63 (2.4%)	183 (1.4%)	1.71 (1.32, 2.21)	<0.0001	1.6 (1.2, 2)	0.0007
Pneumonia (−)	2,565 (97.6%)	12,940 (98.6%)				
Asthma	(N = 4,644)	(N = 23,215)				
Pneumonia (+)	51 (1.1%)	139 (0.6%)	1.8 (1.36, 2.38)	<0.0001	1.6 (1.2, 2.2)	0.0008
Pneumonia (−)	4,593 (98.9%)	23,076 (99.4%)				

Subgroup risk analyses was also conducted according to past history of pneumonia, COPD and asthma. ICS/LABA: Inhaled Corticosteroids/Long acting beta agonist; pMDI: pressurized metered-dose inhaler; DPI: Dry power inhaler; HR: Hazard ratio.

*Adjustment covariates for risk assessment of pneumonia were comorbidities, healthcare utilization, concomitant other medications, and cumulative dose of ICS/LABA usage.

**Hazard ratio was calculated as the ratio of risk of pneumonia in pMDI group to DPI group.

Even when the subgroup was restricted to COPD patients, the risk for pneumonia was higher in the pMDI group (adjusted HR 1.6, [95% CI 1.3–2.0]; p < 0.0001). In asthma patients, the trends were consistent although the incidence of pneumonia was lower than in COPD patients (Table 4).

When the time from initiation of ICS/LABA to pneumonia was compared between two groups, mean duration to pneumonia (±SD) was significantly shorter in pMDI group than in DPI group (69.8 ± 53.6 days vs. 73.9 ± 52.5 days respectively, p = 0.0001). Also pneumonia was developed significantly earlier in pMDI group than in DPI group, when the patients had a history of previous pneumonia (54.5 ± 47.9 days vs. 62.1 ± 49.1 days, p = 0.005), while there was no difference of time to pneumonia in patients without history of pneumonia (77.45 ± 54.6 days vs. 77.8 ± 53.6 days, p > 0.05).

## Discussion

This retrospective cohort study determined the risk for pneumonia in patients using ICS/LABA, which was higher in the pMDI group compared with the DPI group. Regardless of the presence of previous pneumonia and in COPD or asthma patients, the increased risk for pneumonia in the pMDI group was consistent in this study population. Although several previous studies have compared the efficacy and safety of these two devices^13,15,16^, this was the first study to compare the pneumonia risk as a primary outcome according to the type of inhaler device in a large group of new ICS/LABA users.

In previous studies, the incidence of pneumonia was not the primary outcome measure. Moreover, these observational studies, which included less than a few thousand patients, provided no information on pneumonia^16^, or presented an incidence too low to compare statistically^13^ (e.g., 9 of 822 patients in pMDI vs 7 of 822 patients in DPI). On the other hand, we matched a relatively large number of patients (>47,632), which yielded 528 pneumonia patients for meaningful comparison. Overall, the incidence of pneumonia requiring hospitalization was similar to that of this study. In addition, present study tried to minimize bias in the analysis using exact matching process and adjusting covariates which may have affected the risk for pneumonia.

Although the pathophysiological basis explaining the results may be limited, some plausible explanation for the higher risk for pneumonia in pMDI users than DPI user may be inferred.

For inhaled medications, clinical effects and adverse events are associated with the dose deposited in the lung^18^, and there is a dose-response relationship between ICS and pneumonia^6,19^. Although there is controversy regarding the fine particle fraction of pMDIs and DPIs, it may depend on each drug formulation, FP/SAL delivered by pMDI contains a high dose of fine particles, and may be active at lower dose compared with DPI^13,20^.

However, adherence to medication and inhaler techniques are critical contributing factors that could not be assessed in this study. In this view, another plausible point is the risk for overuse of pMDI in relatively short periods compared with DPI. Because overuse of pMDIs compared with single-dose DPIs has been reported^21^, and pneumonia risk associated with ICS is dose-dependent, we cannot ignore the possibility of overuse in the pMDI group, even with adjustment of the the variable. In this study, the mean cumulative dose counts were higher in the pMDI group than in the DPI group, even though the number of prescription days was smaller in the pMDI group (Table 2). The hypothesis may be supported by the finding that the time from initiation of ICS/LABA to pneumonia was shorter in pMDI group than in DPI group. Considering that FP/SAL pMDI has one-half the dose of FP/SAL DPI in usual formulations as a single inhalation (e.g., 125/25 µg FP/SAL as pMDI; 250/50 µg FP/SAL as DPI) and 2 puffs of pMDI were similar dosage for 1 puff of DPI, the median delivery dosage may be suggested to be similar between the two groups. Nevertheless, the patients with higher dose prescription—even in shorter prescription days—were more prevalent in the pMDI group.

Potential weakness to humidity of FP/SAL DPI^22^ and insufficient inspiratory flow rate in inhaling DPI^23,24^, especially in elderly patients, may be a contributing factor increasing the risk for pneumonia in the pMDI group. However, because technical errors in using inhalers are very common among pMDI and DPI users^25^, we could not assess whether poor performance in using inhalers impacts the increase or decrease in the risk for pneumonia. Nevertheless, it should not be overlooked that results of present study were consistent across diseases.

Despite the interesting findings, there are some considerations that should be taken into account before generalizing the results. First, only one FDC ICS/LABA (FP/SAL) was available as a pMDI while two types of DPI (BUD/FOR and FP/SAL) could be prescribed during the study periods (2009–2011) in Korea. Acknowledging reports that FP is more likely to increase pneumonia risk in COPD compared with BUD^26^ and the proportion of FP/SAL DPI was 75.3% of all ICS/LABA DPIs in total population, dilutional effects of BUD in the DPI group could be a possible confounder of the results in this study. Due to internal regulations for HIRA database, however, single head-to-head comparison could not be performed.

Another issue was the imbalance in the number of prescriptions between the two devices. Because the market share of ICS/LABA DPI has been dominant in Korea, before the matching process, >200,000 patients (*i*.*e*., 236,724) were in the DPI group, while only 7,945 patients were in the pMDI group. The single type of the ICS/LABA pMDI was available and the device seemed to be less preferred to DPIs in study period. It also contributed to the discrepancy that Korean national guidelines for COPD and asthma recommended the medications based on their substances rather than type of inhaler or commercial names. Even after the matching process, which is never perfect, a numerical difference—by a factor of close to 30 times—could be a source of bias in comparing the risk for pneumonia.

This investigation had some limitations frequently encountered in retrospective cohort studies and analyses of insurance databases including: accuracy of operational diagnosis of pneumonia based on ICD-10 codes without information regarding radiographic findings or vital signs; limitations in balanced comparisons of identical medications; limited information regarding the dosage in DPI-type inhalers; and lack of medication adherence information related to the discrepancy between the cumulative dose of prescribed medication and actual dose of inhalation.

Despite these limitations, we believe that this real-world study, which included a large sample size (>23,000 patients), and proper matching, could be impactful in clinical practice and lead to—if not at least, inform—further investigations addressing this issue.

In conclusion, through analysis of a national insurance database, new users of pMDI ICS/LABA inhalers demonstrated a higher risk for pneumonia than new users of DPI ICS/LABA inhalers. Further prospective studies comparing the incidence of pneumonia with new inhalers are warranted.

## References

[1] Finney L (2014). Inhaled corticosteroids and pneumonia in chronic obstructive pulmonary disease. Lancet Respir Med..

[2] Qian CJ, Coulombe J, Suissa S, Ernst P (2017). Pneumonia risk in asthma patients using inhaled corticosteroids: a quasi-cohort study. Br J Clin Pharmacol..

[3] Calverley PM (2007). Salmeterol and fluticasone propionate and survival in chronic obstructive pulmonary disease. New England Journal of Medicine..

[4] Vestbo J (2013). Global strategy for the diagnosis, management, and prevention of chronic obstructive pulmonary disease: GOLD executive summary. American journal of respiratory and critical care medicine..

[5] Bateman ED (2008). Global strategy for asthma management and prevention: GINA executive summary. European Respiratory Journal..

[6] Drummond MB, Dasenbrook EC, Pitz MW, Murphy DJ, Fan E (2008). Inhaled corticosteroids in patients with stable chronic obstructive pulmonary disease: a systematic review and meta-analysis. Jama..

[7] Crim C (2009). Pneumonia risk in COPD patients receiving inhaled corticosteroids alone or in combination: TORCH study results. European Respiratory Journal..

[8] Singanayagam A (2018). Corticosteroid suppression of antiviral immunity increases bacterial loads and mucus production in COPD exacerbations. Nat Commun..

[9] Larsson K (2013). Combination of budesonide/formoterol more effective than fluticasone/salmeterol in preventing exacerbations in chronic obstructive pulmonary disease: the PATHOS study. J Intern Med..

[10] Oh YM (2017). Is the Combination of ICS and LABA, a Therapeutic Option for COPD, Fading Away?. Tuberc Respir Dis (Seoul)..

[11] Lavorini F, Pistolesi M, Usmani OS (2017). Recent advances in capsule-based dry powder inhaler technology. Multidiscip Respir Med..

[12] NEWMAN STEPHEN P (1995). A Comparison of Lung Deposition Patterns Between Different Asthma Inhalers. Journal of Aerosol Medicine.

[13] Jones R (2017). The comparative effectiveness of initiating fluticasone/salmeterol combination therapy via pMDI versus DPI in reducing exacerbations and treatment escalation in COPD: a UK database study. Int J Chron Obstruct Pulmon Dis..

[14] Newman S (2005). Inhaler treatment options in COPD. European Respiratory Review..

[15] Koser A, Westerman J, Sharma S, Emmett A, Crater GD (2010). Safety and efficacy of fluticasone propionate/salmeterol hydrofluoroalkane 134a metered-dose-inhaler compared with fluticasone propionate/salmeterol diskus in patients with chronic obstructive pulmonary disease. The open respiratory medicine journal..

[16] Price D (2011). Device type and real-world effectiveness of asthma combination therapy: an observational study. Respir Med..

[17] Austin PC (2008). A critical appraisal of propensity-score matching in the medical literature between 1996 and 2003. Stat Med..

[18] Newman SP (2000). Can lung deposition data act as a surrogate for the clinical response to inhaled asthma drugs?. Br J Clin Pharmacol..

[19] Nannini, L., Cates, C. J., Lasserson, T. J. & Poole, P. Combined corticosteroid and long-acting beta-agonist in one inhaler versus placebo for chronic obstructive pulmonary disease. *Cochrane Database Syst Rev*. CD003794 (2007). 10.1002/14651858.CD003794.pub3 PMC4164185 17943798

[20] Martin RJ (2002). Systemic effect comparisons of six inhaled corticosteroid preparations. Am J Respir Crit Care Med..

[21] Koehorst-ter Huurne K, Movig K, van der Valk P, van der Palen J, Brusse-Keizer M (2016). The influence of type of inhalation device on adherence of COPD patients to inhaled medication. Expert Opin Drug Deliv..

[22] Le VN, Hoang Thi TH, Robins E, Flament MP (2012). Dry powder inhalers: study of the parameters influencing adhesion and dispersion of fluticasone propionate. AAPS PharmSciTech..

[23] Feddah MR, Brown KF, Gipps EM, Davies NM (2000). *In-vitro* characterisation of metered dose inhaler versus dry powder inhaler glucocorticoid products: influence of inspiratory flow rates. J Pharm Pharm Sci..

[24] Rau JL (2006). Practical problems with aerosol therapy in COPD. Respir Care..

[25] Rogliani, P. *et al*. Optimizing drug delivery in COPD: The role of inhaler devices. *Respiratory Medicine*. (2017). 10.1016/j.rmed.2017.01.006 28284323

[26] Janson C (2013). Pneumonia and pneumonia related mortality in patients with COPD treated with fixed combinations of inhaled corticosteroid and long acting β2 agonist: observational matched cohort study (PATHOS). Bmj..

